# Anti-Müllerian hormone, progesterone, and estrogen at breeding season onset and their association with reproductive performance in multiparous ewes

**DOI:** 10.3389/fendo.2026.1946907

**Published:** 2026-08-26

**Authors:** Abdurrahman Takci, Tahsin Takci

**Affiliations:** 1 Department of Obstetrics and Gynecology, Faculty of Veterinary Medicine, Sivas Cumhuriyet University, Sivas, Türkiye; 2 Department of Obstetrics and Gynecology, Sivas Numune Hospital, Sivas, Türkiye

**Keywords:** anti-Müllerian hormone, estrogen, ewe, ovarian reserve, progesterone, reproductive performance

## Abstract

**Background:**

Ovarian reserve is established during fetal life and progressively declines with age, a process accompanied by increased follicular atresia, impaired steroidogenic capacity of granulosa cells, and consequent alterations in circulating anti-Müllerian hormone (AMH), estradiol, and progesterone levels. These changes may compromise ovulatory efficiency, uterine receptivity and luteal function, thereby influencing conception rates and the risk of early pregnancy loss. Because ewes combine a long reproductive lifespan with follicular dynamics that resemble those of women, they are of interest as a large-animal model of ovarian ageing; in women, declining follicular reserve and falling AMH are established markers of reduced fertility. This study evaluated the association between serum AMH, progesterone and estrogen concentrations measured at the onset of the breeding season and subsequent reproductive performance in multiparous ewes.

**Methods:**

Forty-four multiparous kangal ewes aged 6–8 years, with a history of multiple prior parturitions, were used as study material. Blood samples were collected at the onset of the breeding season to determine serum AMH, estrogen, and progesterone concentrations. Animals were monitored throughout the breeding season for time to conception and pregnancy outcome, and were subsequently allocated into six groups: Group 1 (n=8, conceived in the 1st cycle), Group 2 (n=7, conceived in the 2nd–3rd cycle), Group 3 (n=5, conceived in the 2nd–3rd cycle but experienced pregnancy loss), Group 4 (n=8, conceived in the 4th cycle), Group 5 (n=8, conceived in the 5th–8th cycle), and Group 6 (n=8, failed to conceive throughout the breeding season). Hormone concentrations were compared among groups, and Spearman correlation analysis was performed to assess pairwise hormonal relationships.

**Results:**

Serum AMH concentrations were significantly higher in Group 1 compared with Group 3 (P<0.01), Group 5 (P<0.01), and Group 6 (P<0.05), and in Group 2 compared with Group 3 (P<0.01) and Group 5 (P<0.01). No significant differences in AMH were observed between the remaining group pairs. Serum progesterone and estrogen concentrations did not differ significantly among groups (P>0.05). Across the entire cohort, progesterone and estrogen demonstrated a moderate positive correlation (Spearman’s ρ = 0.387), indicating that animals with higher progesterone concentrations tended to have higher estrogen concentrations. This association remained significant after Benjamini-Hochberg correction within the whole-cohort correlation family (P = 0.010; q = 0.030), whereas no significant correlation was detected between AMH and progesterone (ρ = -0.248, P = 0.104) or between AMH and estrogen (ρ = -0.146, P = 0.346). None of the exploratory within-group correlations survived correction for multiple testing.

**Conclusions:**

Among the three hormones evaluated, AMH was the only one that differed significantly among reproductive outcome groups; progesterone and estrogen showed no significant differences, although the study was powered to detect only large effects and smaller but biologically meaningful differences cannot be excluded. These findings are consistent with the proposed value of the ewe as a model of human ovarian ageing, a parallel that currently rests on phenotypic rather than established mechanistic similarity, and indicate that AMH measured before mating is associated with subsequent reproductive performance in aged multiparous ewes. Prospective longitudinal studies using species-specific cohorts are warranted to better characterize the timeline and underlying mechanisms of ovarian aging.

## Introduction

Reproductive efficiency constitutes one of the primary determinants of profitability and long-term sustainability in sheep production systems worldwide (1). While considerable research attention has been directed toward the reproductive management of young ewes and ewe lambs, the endocrine determinants of reproductive performance in older, multiparous ewes remain comparatively underexplored. In mature flocks, ewes aged six years and above represent a segment of the population that has already demonstrated sustained reproductive capacity; however, age-related changes in ovarian reserve and steroidogenic function may progressively compromise their fertility, introducing a need for reliable endocrine markers capable of identifying individuals at risk of reproductive failure.

The hypothalamic-pituitary-gonadal (HPG) axis regulates the complex interplay of hormones governing ovarian cyclicity, folliculogenesis, and luteal function in ewes (2). Among the endocrine markers relevant to ovarian reserve assessment, Anti-Müllerian Hormone (AMH) has attracted particular interest across species. Secreted exclusively by granulosa cells of small growing antral follicles, AMH reflects the size of the primordial follicle pool and is considered the most reliable endocrine indicator of ovarian reserve currently available (3). In sheep, Lahoz et al. (4) demonstrated that plasma AMH concentration in prepubertal ewe lambs was positively correlated with ovulatory response to eCG stimulation and fertility at first mating, with ewes carrying AMH concentrations above 97 pg/mL achieving conception rates 34.8 percentage points higher than those with lower values. More recently, Turgut and Koca (5) reported that serum AMH levels were positively associated with litter size in Romanov ewes, with higher AMH concentrations (>320 pg/mL) corresponding to larger litters. However, the predictive utility of AMH in older, multiparous ewes remains contested. Acharya et al. (6) reported that AMH concentration was not a reliable predictor of fertility in multiparous Katahdin ewes, highlighting the possibility that the relationship between AMH and reproductive performance may be modulated by parity and age. In a comparative study of two Slovenian sheep breeds, Šterbenc et al. (7) found no significant age-related differences in AMH within the Jezersko–Solčava breed across age groups of 1–3, 4–6, and ≥7 years, though AMH levels remained positively correlated with litter size in ewes over seven years of age, suggesting that AMH may retain predictive value in older animals despite overall follicular reserve decline.

Progesterone, secreted mainly by the corpus luteum after ovulation, establishes and maintains pregnancy by acting on uterine receptivity and embryo implantation (8). Deviations during the periovulatory and early luteal phases have been linked to early embryonic loss in small ruminants: in ewes followed through successive pregnancies, embryo survival was related to progesterone concentrations around ovulation and to the timing and rate of the rise to luteal values (1, 9). Luteal function is also vulnerable to long-term influences. Long et al. (10) found that ewes born to nutrient-restricted mothers tended to have lower circulating and luteal progesterone at six years of age (P = 0.06 and P = 0.07) alongside significantly reduced luteal StAR and P450scc expression (P < 0.05). In aged multiparous ewes, in which cumulative reproductive events and declining corpus luteum function may further alter luteal steroidogenesis, a basal progesterone concentration measured at the onset of the breeding season therefore indicates both cyclicity status and luteal competence, parameters likely to be more heterogeneous in older animals.

Estrogen, predominantly estradiol-17β, governs estrous behavior, triggers the preovulatory LH surge, and modulates uterine receptivity in ewes (11). As ewes mature, the increasing frequency of GnRH and LH pulses associated with declining photoperiod promotes enhanced estradiol synthesis by ovarian follicles, which is considered necessary for the expression of full behavioral estrus and coordinated ovulation (12). In mature cyclic ewes, estradiol secretion from dominant follicles peaks around the time they reach maximum diameter, coupling follicular maturation tightly with the timing of the preovulatory LH surge (13). In older ewes, alterations in follicular dynamics and granulosa cell responsiveness may produce variability in estradiol output at the transition into the breeding season, potentially influencing the probability of fertile mating. Despite its well-established role in ovine reproductive physiology, estradiol has rarely been examined as a predictive biomarker for pregnancy rate in multiparous ewes evaluated at the start of the breeding season.

Sheep are short-day seasonally polyestrous breeders, with the transition from anestrus to cyclic ovarian activity triggered by the decline in photoperiod following the summer solstice (14). Decreasing daylength stimulates melatonin secretion from the pineal gland, which activates hypothalamic GnRH release via the HPG axis and reinstates pulsatile gonadotropin secretion, ultimately initiating follicular development and ovulation (15). The period immediately surrounding the onset of the breeding season is characterized by pronounced individual variability in hormonal profiles, as ewes transition at different rates from anovulatory quiescence to full cyclic activity. In multiparous ewes aged six years and above, this transitional phase may be further complicated by cumulative age-related changes in ovarian follicle populations, luteal function, and hypothalamic sensitivity to steroid feedback, making the characterization of hormone profiles at this juncture of particular relevance.

Aged multiparous ewes are of practical importance because they represent the stage of the productive lifespan at which culling and replacement decisions are routinely made. In commercial flocks these decisions are usually taken retrospectively, after an animal has already failed to conceive or has lost a pregnancy, and therefore after a full breeding season of feed, labor and ram capacity has been committed to her (16–18). A marker that could be measured before mating and that reflected the functional state of the ovary rather than chronological age alone would allow such decisions to be informed prospectively. Failure to conceive is a consistent trigger for removal from the flock, and ewe wastage of this kind carries measurable costs in flock productivity and farm profitability. This is biologically plausible, because ewes of identical age and parity may nonetheless differ substantially in the size of their remaining follicular pool, and it is this inter-individual heterogeneity in ovarian reserve, rather than age per se, that is expected to determine reproductive outcome in mature flocks.

The three hormones examined here differ in a way that is central to the design of the study. AMH is secreted by the granulosa cells of small growing follicles and, in post-pubertal ewes, its circulating concentration is largely independent of the stage of the estrous cycle and comparable in and out of season, so that a single sample can reasonably represent an individual’s status (5, 22). Progesterone and estradiol, by contrast, are intrinsically cyclical: their concentrations at any moment reflect the position of the animal within her cycle rather than a cumulative property of the ovary. A single pre-mating sample is therefore expected *a priori* to be informative for AMH but not necessarily for the two steroids, and this distinction was anticipated when the sampling strategy was chosen rather than invoked afterwards to explain the results. Accordingly, the present study was designed to determine the extent to which serum AMH, progesterone, and estrogen concentrations assessed at the onset of the breeding season are associated with subsequent conception timing and pregnancy outcome in multiparous ewes.

## Materials and methods

### Animals and study design

This study was conducted during a single breeding season at the sheep production unit of Erka Tarım Hayvancılık Farm, located in Ortaklar Village, Yıldızeli District, Sivas Province, Türkiye (39.861° N, 36.349° E; 1,310 m above sea level). The study area lies in the Central Anatolian highlands and has a continental climate, with long, cold and snowy winters and hot, dry summers; owing to the relatively high altitude the growing season is short and forage production depends largely on seasonal precipitation. Forty-four multiparous Kangal ewes aged 6–8 years were used; all animals were of the same breed. At the onset of the study, the body condition score (BCS) of the ewes ranged from 2.50 to 3.25 on a 0–5 scale (**Table 1**). Blood samples were collected at the beginning of the breeding season (1 September), and serum concentrations of anti-Müllerian hormone (AMH), estrogen and progesterone were determined.

**Table 1:** Body weight, body condition score and parity of the groups classified according to reproductive performance, measured at the onset of the breeding season (mean ± SD).

Group	n	Body weight (kg)	Body condition score	Parity
Group 1 – Conceived at the first cycle	8	58.29 ± 2.70	2.84 ± 0.23	5.25 ± 0.89
Group 2 – Conceived at cycles 2–3	7	58.40 ± 2.64	2.89 ± 0.28	5.14 ± 0.90
Group 3 – Conceived at cycles 2–3, with subsequent pregnancy loss	5	58.82 ± 3.44	2.85 ± 0.29	4.80 ± 0.84
Group 4 – Conceived at cycle 4	8	58.59 ± 2.55	2.88 ± 0.23	5.25 ± 0.89
Group 5 – Conceived at cycles 5–8	8	58.01 ± 2.53	2.84 ± 0.23	5.00 ± 0.93
Group 6 – Failed to conceive throughout the breeding season	8	58.30 ± 2.59	2.97 ± 0.25	5.13 ± 0.83
**Overall**	**44**	**58.37 ± 2.55**	**2.88 ± 0.24**	**5.11 ± 0.84**
*p*		0.996	0.915	0.934

SD, standard deviation. Values are presented as mean ± standard deviation. Forty-four multiparous ewes aged 6–8 years were used; the animals were exposed to natural mating throughout the breeding season (8 cycles, approximately 140 days) and were subsequently allocated to six groups according to their reproductive performance. Measurements were taken at the onset of the breeding season (1 September). Body condition score was assessed on a 0–5 scale. Parity denotes the total number of previous lambings. Body weight and body condition score were compared among groups by one-way ANOVA (F (5,38) = 0.069 and F (5,38) = 0.291, respectively); as parity is a count variable, the Kruskal–Wallis test was used (H = 1.312). No statistically significant difference was detected among the groups for any variable (*p* > 0.05), indicating that the groups were balanced for body weight, body condition and parity at the onset of the study. Bold type denotes the pooled values for the whole cohort and the P values for the comparison among the six groups.

The experimental ewes were maintained within a flock comprising 900 ewes and 60 rams, in which natural mating was permitted continuously throughout the breeding season. The rams used were of proven fertility, aged 3–5 years, weighing 102–117 kg, with a BCS of 3.00–3.25. All animals were kept under identical management and feeding conditions and were exposed to natural service by the 60 fertile rams throughout the entire breeding season.

The ewes were monitored over eight consecutive estrous cycles (approximately 140 days) and subsequently until the outcome of pregnancy was determined. Based on the reproductive performance recorded during this period, the ewes were allocated to six groups: Group 1 (n = 8), ewes that conceived at the first cycle; Group 2 (n = 7), ewes that conceived at the second or third cycle and carried the pregnancy to term; Group 3 (n = 5), ewes that conceived at the second or third cycle but subsequently experienced pregnancy loss (abortion, n = 2; premature parturition, n = 3); Group 4 (n = 8), ewes that conceived at the fourth cycle; Group 5 (n = 8), ewes that conceived between the fifth and eighth cycle; and Group 6 (n = 8), ewes that failed to conceive throughout the breeding season. Body weight, BCS and parity of the groups at the onset of the breeding season are presented in **Table 1**. The hormonal profiles obtained at the beginning of the breeding season were then evaluated in relation to the reproductive performance of the ewes.

### Feeding and pasture evaluation

Throughout the study, all experimental groups grazed the same natural pasture for approximately 10–12 h per day. In addition to grazing, the ewes received a total of 300 g of ground barley per head per day, offered in two equal meals (2 × 150 g) in the morning and on return from pasture in the evening. This ration formed part of the routine feeding program applied on the farm throughout the year; no change was made before or after the breeding season, and no additional energy supplementation (flushing) specific to the breeding season was provided. Water was offered three times at 3–4 h intervals while the animals were at pasture and was available ad libitum after their return.

To determine the nutrient composition of the pasture, herbage samples were collected with shears from five randomly selected points within the area most intensively grazed by the animals, cutting approximately 2–3 cm above ground level. Dry matter (DM), organic matter (OM), crude protein (CP), ether extract (EE) and crude ash contents were determined according to the methods described by AOAC (19). Neutral detergent fiber (NDF) and acid detergent fiber (ADF) contents were analyzed using an Ankom 200 Fiber Analyzer (Ankom Technology Corp., Fairport, NY, USA) following the procedure of Van Soest et al. (20). The nutrient composition of the pasture is presented in **Table 2**.

**Table 2:** Nutrient composition of the pasture used in the study.

Item	Content
Dry matter (%, as fed)	82.66
Organic matter (% DM)	89.77
Crude protein (% DM)	9.56
Ether extract (% DM)	2.55
Crude ash (% DM)	10.23
aNDF (% DM)	64.50
ADF (% DM)	40.20

DM, dry matter; aNDF, neutral detergent fiber assayed with heat-stable α-amylase; ADF, acid detergent fiber. All values except dry matter are expressed on a dry matter basis.

### Pregnancy diagnosis

The mating date of each ewe was recorded. To identify the cycle at which conception occurred, pregnancy examinations were performed by transrectal ultrasonography between days 18 and 22 after mating throughout the breeding season. Prior to examination, the ewes were restrained in dorsal recumbency, and scanning was carried out using a portable ultrasound device (Mindray DP-10, Mindray Bio-Medical Electronics Co., Ltd., Shenzhen, China) equipped with a 7.5 MHz linear probe. Pregnancy was diagnosed on the basis of the presence of an embryonic vesicle, fetal membranes, an embryo/fetus and fetal heartbeat. Ewes not diagnosed as pregnant at the first examination were re-mated during subsequent cycles and re-examined using the same protocol, so that the cycle at which conception occurred could be determined unequivocally.

In ewes diagnosed as pregnant, a second examination was performed transabdominally with a convex probe at approximately day 60 of gestation. Pregnancies were subsequently monitored until parturition through regular farm visits, and abortions, premature parturitions and normal lambings were recorded. Body weight and body condition score were recorded for every ewe at the onset of the breeding season, and did not differ significantly among the outcome groups at that point (**Table 1**). All animals were kept as a single management group on the same pasture and received the same supplementary barley ration throughout the study, so that nutritional treatment was uniform by design; no further metabolic profiling, such as blood urea nitrogen, was undertaken, and this is acknowledged as a limitation.

### Blood sampling, serum preparation and analysis

Blood samples (approximately 8 mL) were collected from each ewe by jugular venipuncture into plain serum tubes without anticoagulant. Samples were allowed to clot at room temperature for 30–60 min and were subsequently centrifuged at 1,500 × g for 10 min at 4 °C. The separated serum was transferred into sterile microtubes and stored at −80 °C until analysis; samples were thawed only once.

Serum anti-Müllerian hormone (AMH) concentrations were measured using a commercially available electrochemiluminescence immunoassay (ECLIA) (Elecsys AMH, Roche Diagnostics GmbH, Mannheim, Germany) on a cobas e immunoassay analyzer. The assay demonstrated excellent analytical precision, with a repeatability (intra-assay coefficient of variation, CV) ranging from 0.8% to 2.1% and an intermediate precision (inter-assay CV) ranging from 2.8% to 3.8%, depending on analyte concentration. Serum progesterone concentrations were determined using the Elecsys Progesterone III assay (Roche Diagnostics GmbH, Mannheim, Germany; catalog no. 07092539190), with a measuring range of 0.05–60 ng/mL and a limit of detection of 0.05 ng/mL. Serum estradiol concentrations were measured using the Elecsys Estradiol III assay (Roche Diagnostics GmbH, Mannheim, Germany). All hormone analyses were performed according to the manufacturer’s instructions using the same analytical platform throughout the study. 

### Statistical analysis

An *a priori* sample size calculation was performed using G*Power version 3.1 (21) for a one-way fixed-effects ANOVA. Assuming a large effect size (Cohen’s *f* = 0.68), a significance level of α = 0.05, statistical power (1 − β) = 0.90, and six comparison groups, the minimum required total sample size was calculated as 42 animals; 44 ewes were enrolled. This effect size was adopted on pragmatic grounds, reflecting the number of aged multiparous ewes of uniform age and parity available within a single flock, rather than being derived from previously published between-group differences in ovine AMH, for which no directly comparable estimate exists. Because this assumption restricts the study to the detection of large effects, a sensitivity analysis was performed for the realized design (N = 44, six groups, α = 0.05): the smallest detectable effect was Cohen’s f = 0.58 at 80% power and f = 0.66 at 90% power, corresponding to η² of approximately 0.25 and 0.30. Differences smaller than this could not have been detected reliably, and non-significant findings are interpreted accordingly throughout.

Data normality was assessed using the Kolmogorov–Smirnov test. Since the data did not follow a normal distribution, non-parametric tests were applied. Differences among multiple independent groups were evaluated using the Kruskal–Wallis test. When significant overall differences were detected, pairwise comparisons were performed using Dunn’s *post-hoc* test, for which GraphPad Prism reports multiplicity-adjusted P values based on the Bonferroni–Dunn procedure; the adjusted P values are those quoted throughout, and significance was assessed against the conventional threshold of 0.05. Results are expressed as median and interquartile range (IQR; 25th–75th percentiles). A P value of < 0.05 was considered statistically significant. All statistical analyses were performed using GraphPad Prism (version 8.0.1; GraphPad Software, San Diego, CA, USA). All hormone concentrations are presented on the original measurement scale; no logarithmic or other transformation was applied. No formal outlier detection procedure was used and no observation was excluded from any analysis, since the rank-based tests employed here are insensitive to extreme values.

Spearman rank correlation analysis was additionally performed within each study group to explore hormone interrelationships in specific clinical outcome subgroups. Due to the limited sample sizes within individual groups (n = 5–8), within-group analyses were considered exploratory. Spearman correlation coefficients (ρ) were interpreted primarily as measures of effect size and association strength, whereas P values were regarded as supportive indicators of statistical evidence. Accordingly, these subgroup findings were interpreted with appropriate caution. All correlation coefficients are reported as Spearman’s ρ together with their corresponding two-tailed P values. Because 21 correlation tests were computed in total, the Benjamini–Hochberg procedure was applied to control the false discovery rate. Correction was performed separately within two pre-defined families: the three whole-cohort correlations, which constituted the pre-specified analysis, and the 18 within-group correlations, which were exploratory. Both nominal P values and Benjamini–Hochberg adjusted values (q) are reported. A P value of < 0.05 was considered statistically significant.

## Results

**Figure 1:**
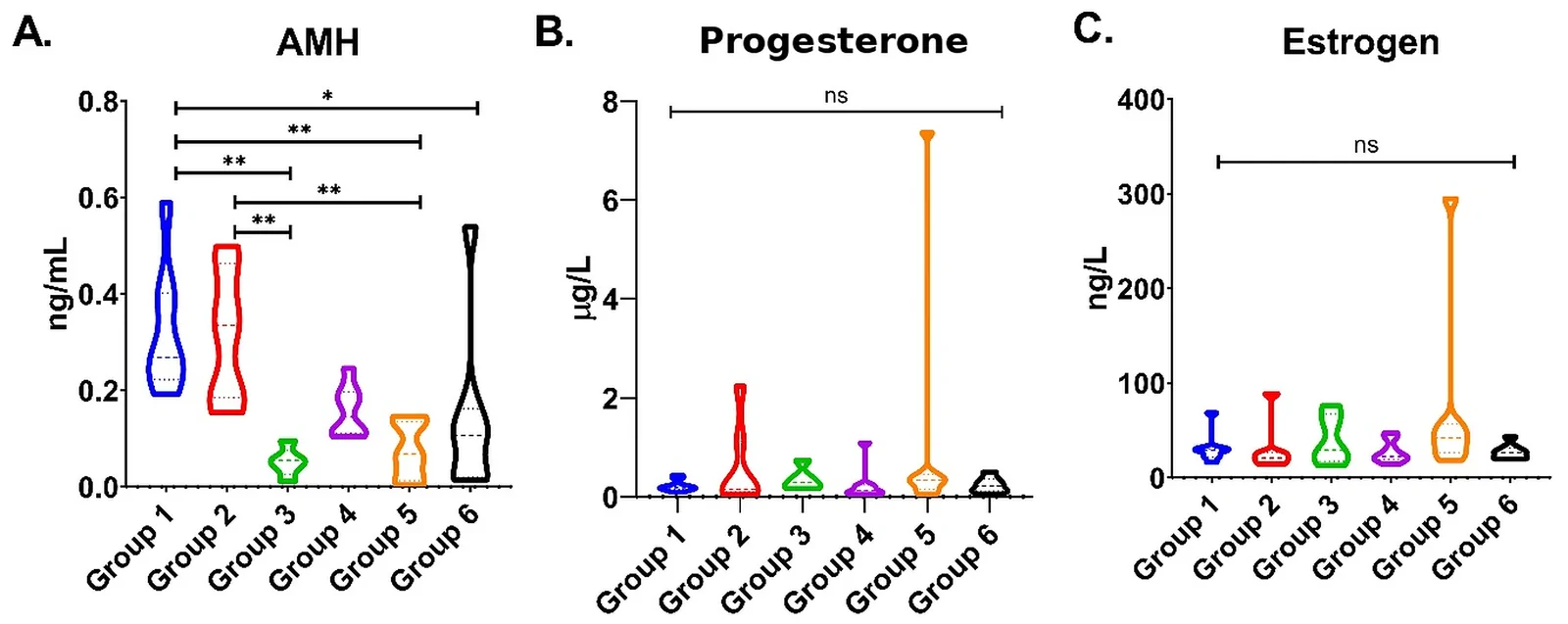
Serum hormone levels among groups. Violin plots illustrating the distribution of serum **(A)** anti-Müllerian hormone (AMH; ng/mL), **(B)** progesterone (µg/L), and **(C)** estrogen (ng/L) concentrations across six groups: Group 1 (n = 8, conceived in the 1st cycle), Group 2 (n = 7, conceived in the 2nd–3rd cycle), Group 3 (n = 5, conceived in the 2nd–3rd cycle but experienced pregnancy loss), Group 4 (n = 8, conceived in the 4th cycle), Group 5 (n = 8, conceived in the 5th–8th cycle) and Group 6 (n = 8, failed to conceive throughout the breeding season). Dashed lines within each violin represent the median and interquartile range (25th–75th percentiles). Data are shown on the original measurement scale; no transformation was applied and no observation was excluded as an outlier. Kolmogorov–Smirnov test, Kruskal–Wallis test and Dunn’s post-hoc multiple comparison test. *P < 0.05; **P < 0.01; ns, not significant.

Serum AMH, progesterone, and estrogen levels were compared across the six study groups. Kruskal–Wallis analysis revealed a statistically significant difference in AMH concentrations among groups (P < 0.05). Subsequent pairwise analysis using Dunn’s *post-hoc* test demonstrated that Group 1 (1st-cycle pregnancies) had significantly higher AMH levels compared to Group 3 (2nd–3rd cycle pregnancies with pregnancy loss; P < 0.01), Group 5 (5th–8th cycle pregnancies; P < 0.01), and Group 6 (no pregnancy throughout the breeding season; P < 0.05). Similarly, Group 2 (2nd–3rd cycle pregnancies) exhibited significantly higher AMH levels than Group 3 (P < 0.01) and Group 5 (P < 0.01). No statistically significant differences in AMH were observed between the remaining group pairs. Group medians and interquartile ranges for all three hormones are displayed in **Figure 1**.

In contrast, serum progesterone levels did not differ significantly across groups, as determined by Kruskal–Wallis analysis (P > 0.05). Likewise, serum estrogen concentrations showed no statistically significant variation among the six groups (P > 0.05). Median (IQR) progesterone and estrogen concentrations are given in **Figures 1B, C**. Although a small number of ewes in Group 5 showed markedly higher progesterone and estrogen values than the remainder of the cohort, these observations did not shift the group median, and the rank-based Kruskal–Wallis test is by construction insensitive to their magnitude; the group medians for both steroids therefore remained closely comparable across the six groups. No observation was excluded on the grounds of being extreme. Accordingly, AMH was the only one of the three hormones for which a statistically significant difference among outcome groups could be demonstrated in this cohort. Because the design was powered to detect only large effects (see Statistical Analysis), this result should be read as an absence of detectable differences in progesterone and estrogen under the present sampling strategy and sample size, rather than as evidence that these hormones lack discriminatory value.

**Figure 2:**
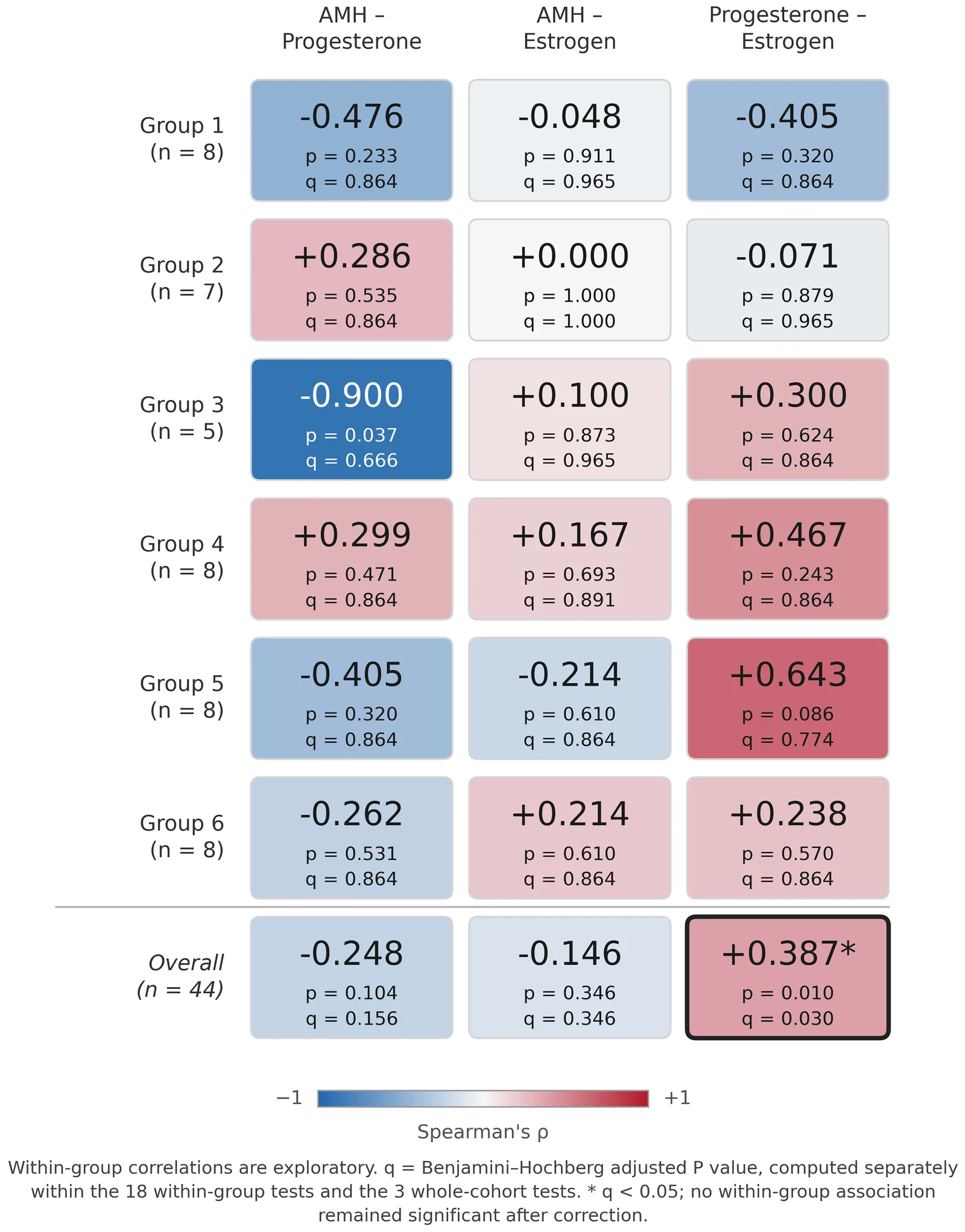
Within-group Spearman correlation matrix. Heatmap illustrating pairwise Spearman correlation coefficients (ρ) between serum anti-Müllerian hormone (AMH), progesterone, and estrogen concentrations, calculated separately for each of the six study groups and for the overall cohort (n = 44). Rows represent study groups (Group 1–6) and the overall sample; columns represent the three hormone pairs (AMH–Progesterone, AMH–Estrogen, and Progesterone–Estrogen). Cell color reflects the direction and magnitude of ρ: blue indicates a negative correlation, red indicates a positive correlation, and white indicates no correlation. Spearman’s ρ and corresponding nominal p values are displayed within each cell. Benjamini–Hochberg adjusted values (q) are shown beneath each nominal P value and were computed separately within the 18 within-group tests and the 3 whole-cohort tests. All within-group analyses are exploratory and none remained significant after correction (smallest q = 0.666); no within-group cell is therefore marked with an asterisk or an emphasized border. The only association surviving correction is the whole-cohort progesterone–estrogen correlation (q = 0.030), which is marked accordingly. *q < 0.05 after Benjamini-Hochberg correction. Group 3: ewes who conceived in the 2nd–3rd cycles but experienced pregnancy loss (n = 5).

Within-group Spearman correlation analysis was performed to explore pairwise hormone associations separately for each clinical outcome subgroup (**Figure 2**). Across the overall cohort (n = 44), serum progesterone and estrogen levels showed a statistically significant positive correlation (*ρ = 0.387, p = 0.010*; Benjamini–Hochberg q = 0.030), whereas no significant associations were observed between AMH and progesterone (*ρ = −0.248, p = 0.104*) or AMH and estrogen (*ρ = −0.146, p = 0.346*).

When analyzed at the group level, the majority of within-group correlations did not reach statistical significance, likely reflecting the limited statistical power of the small subgroup sizes (n = 5–8). Within Group 3 (n = 5), a very strong negative association was observed between AMH and progesterone (Spearman’s ρ = −0.900). Although this correlation reached nominal statistical significance (P = 0.037), it did not survive Benjamini–Hochberg correction across the 18 exploratory within-group tests (q = 0.67), and the very small sample size warrants cautious interpretation. No within-group correlation remained significant after correction for multiple testing. Therefore, the magnitude of the correlation coefficient should be considered more informative than the P value, and this finding should be regarded as exploratory and hypothesis-generating rather than confirmatory. No other within-group pair reached significance. Notably, in Group 5 (5th–8th cycle pregnancies), a moderate positive trend was observed between progesterone and estrogen (*ρ = 0.643, p = 0.086*), though this did not reach statistical significance.

## Discussion

This study was designed to characterize ovarian aging in ewes of advanced reproductive age and to determine whether hormonal markers measured at the onset of the breeding season reflect the reproductive consequences of that aging. Its central finding is that, among the three hormones evaluated, only serum anti-Müllerian hormone (AMH) discriminated among clinical reproductive outcomes. Ewes that conceived in the first cycle (Group 1) and those that conceived within the second to third cycles (Group 2) had significantly higher AMH concentrations than ewes that experienced pregnancy loss (Group 3), those that conceived only late in the season (Group 5), and those that failed to conceive altogether (Group 6). By contrast, neither progesterone nor estradiol differed significantly among groups, and neither was correlated with AMH. A key implication is that even within a population of old ewes that was homogeneous in both chronological age (six to eight years) and parity, circulating AMH revealed substantial inter-individual variation in the functional ovarian reserve, and this variation—an index of biological “ovarian age” that is not captured by chronological age or reproductive history alone—tracked closely with subsequent fertility. Pre-mating AMH therefore emerges as a minimally invasive marker of the reproductive impact of ovarian aging in high-parity animals, extending to a mature ovine population a relationship established largely in young ewes and in cattle.

The discriminative value of AMH observed here is biologically coherent. AMH is secreted almost exclusively by the granulosa cells of small growing preantral and antral follicles, and circulating concentrations are positively associated with the antral follicle count, making AMH the most reliable endocrine index of the functional ovarian reserve currently available in ruminants (22, 23). A larger pool of gonadotropin-responsive follicles at the transition from anestrus to cyclicity plausibly translates into earlier resumption of ovulatory cycles and more efficient conception once mating begins, which is consistent with the higher AMH concentrations we found in the early-conceiving groups. The approach also rests on a methodological premise that recent ovine work has strengthened: because AMH concentrations in post-pubertal ewes remain stable across the estrous cycle and between in-season and off-season time points, a single measurement can reliably reflect an individual’s circulating AMH status (5, 24). This cycle-independence is precisely what makes a one-off, season-onset AMH sample informative under field conditions, and it distinguishes AMH from the steroid hormones discussed below.

Our findings are concordant with a substantial body of evidence linking the ovarian reserve, as indexed by AMH, to reproductive performance across ruminants. The positive association between circulating AMH and antral follicle number is well established, with higher values repeatedly related to superior reproductive outcomes (22, 23). In sheep specifically, Lahoz et al. (4) demonstrated that prepubertal Rasa Aragonesa ewe lambs with higher plasma AMH ovulated more readily following eCG stimulation and achieved markedly higher conception rates at first mating, establishing AMH as an individually stable marker of ovarian competence early in life. This relationship appears to persist into adulthood and to extend from the probability of conception to litter size: Turgut and Koca (5) reported that Romanov ewes with higher serum AMH produced larger litters, and Šterbenc et al. (7) found that, although AMH did not decline significantly across age strata in the Jezersko–Solčava breed, it nonetheless remained positively associated with litter size in ewes older than seven years—evidence that AMH can retain predictive value even in advanced age. Our data are consistent with this trajectory and extend it to a population in which the predictive utility of AMH has been least examined: aged, high-parity ewes evaluated prospectively against their realized reproductive outcome within a single breeding season.

These concordances notwithstanding, the predictive utility of AMH in adult ewes is not universally supported, and our results must be weighed against discrepant findings. Most directly, Acharya et al. (6) reported that circulating AMH did not predict fertility in multiparous Katahdin ewes, a population superficially comparable to our own. Several methodological and biological considerations may reconcile this divergence. First, breed exerts a strong influence on both the absolute AMH set-point and intrinsic prolificacy, with reported mean concentrations differing severalfold between prolific and non-prolific breeds (7, 25) a signal that is detectable within one breed may therefore be obscured in another whose fertility is constrained by different factors. Second, absolute AMH values and the position of any predictive threshold are assay-dependent, and the absence of standardized, species-specific reference ranges for ovine AMH hampers direct cross-study comparison. Third, and arguably most important, the definition of the reproductive endpoint differs across studies: a binary pregnant/non-pregnant classification may lack the resolution to detect a graded relationship with a continuous underlying variable, whereas our stratification across first-cycle conception, delayed conception, pregnancy loss, and reproductive failure affords greater sensitivity to the ovarian-reserve gradient. Fourth, realized fertility is inherently multifactorial—uterine receptivity, luteal competence, nutritional status, ram effects, and management all contribute—so in cohorts where non-ovarian factors predominate, the ovarian-reserve signal carried by AMH may be masked. The uniform chronological age and parity of our cohort, together with its constrained body condition, plausibly attenuated several of these competing sources of variation and allowed the contribution of the ovarian reserve to emerge. Interpreted in this light, the apparent conflict in the literature may reflect differences in what each study was methodologically positioned to detect rather than a fundamental inconsistency in the biology of AMH; on balance, the current evidence, including the present study, supports AMH as an informative—though not deterministic—marker of reproductive potential in mature ewes.

A particularly notable aspect of our findings is that low AMH was associated not only with delayed conception but also with pregnancy loss: Groups 1 and 2 had significantly higher AMH than Group 3, in which ewes conceived but subsequently aborted or delivered prematurely. This is consistent with an emerging view that AMH reflects oocyte and embryo quality in addition to follicular quantity; ewe lambs with fewer antral follicles yield oocytes of lower developmental competence (22), and diminished ovarian reserve may therefore compromise not only the probability of conception but also the developmental viability of the resulting conceptus. Direct support comes from recent ovine data indicating that higher AMH concentrations in early pregnancy are associated with fetal viability, whereas lower AMH accompanies fetal death (26). Within Group 3 we additionally observed a strong negative correlation between AMH and progesterone; however, this subgroup comprised only five animals, the analysis was pre-specified as exploratory, and the finding should be regarded as hypothesis-generating rather than confirmatory. It nonetheless raises the possibility that a dysregulated relationship between the follicular reserve and luteal steroidogenesis contributes to early pregnancy failure in aged ewes, a hypothesis that warrants testing in adequately powered cohorts.

The absence of significant differences in progesterone and estradiol among the outcome groups is itself informative and, rather than weakening the rationale for endocrine profiling, clarifies why AMH specifically carried the predictive signal. Unlike AMH, whose concentration is largely independent of cycle stage in post-pubertal ewes and can therefore be captured reliably by a single sample (5, 24), the ovarian steroids are intrinsically dynamic, and their circulating concentrations depend heavily on the exact physiological moment of sampling. At the onset of the breeding season, ewes are emerging from seasonal anestrus at individually variable rates, and during this transition progesterone falls to basal or near-undetectable concentrations because functional corpora lutea are largely absent (2). A single pre-mating progesterone value therefore reflects an animal’s instantaneous cyclicity status rather than its integrated luteal competence across the ensuing season, so its failure to distinguish ewes that would ultimately differ in conception timing or pregnancy maintenance is unsurprising. This does not imply that progesterone is uninformative per se: measured at biologically appropriate windows, deviations in periovulatory and early-luteal progesterone have been linked to early embryonic loss in small ruminants (27), and progesterone profiles during established gestation can flag fetal death (28). The null result obtained here is thus a consequence of the single, season-onset sampling scheme rather than evidence against a physiological role for progesterone.

Comparable reasoning applies to estradiol. Estradiol is secreted transiently and in pulsatile fashion during the brief follicular phase, peaking as the dominant follicle approaches maximal diameter immediately before the preovulatory LH surge (12, 13). A single sample obtained before mating is therefore poorly positioned to capture between-animal differences of prognostic relevance, and any signal is further blurred by the asynchronous resumption of cyclicity across the cohort. The significant positive correlation we observed between progesterone and estradiol across the full sample (ρ = 0.387) is physiologically coherent, reflecting the coordinated follicular and luteal steroidogenesis that accompanies cyclic ovarian activity; yet this shared dependence on momentary cycle status is precisely what limits the prognostic value of either steroid when assessed at a single arbitrary time point. Taken together, these considerations reinforce the central inference of the study: among the three hormones examined, only AMH combines biological relevance to the ovarian reserve with the temporal stability required for single-sample prediction, which is why it alone discriminated reproductive outcomes in this population of aged ewes.

These observations speak directly to the study’s central aim of characterizing ovarian aging in mature ewes and reinforce the value of the small-ruminant model in this field. The sheep and goat are increasingly recognized as tractable large-animal models of ovarian ageing, offering a long reproductive lifespan, seasonal cyclicity, and follicular dynamics that more closely approximate those of women than do rodent models (29). Our data add a functional, outcome-linked dimension to that model: rather than documenting ovarian aging only through cross-sectional age comparisons or post-mortem follicle counts, we show that variation in the ovarian reserve among similarly aged old ewes—their divergent “ovarian ages”—maps onto tangible differences in conception timing and pregnancy maintenance within a single season. The endocrine signature of declining follicular reserve, and the emergence of AMH as its early marker, closely parallels observations in women, in whom pretreatment AMH predicts loss of ovarian function and diminished reproductive potential more reliably than chronological age or gonadotropins (30, 31). This concept—that biological ovarian age can diverge markedly from chronological age and that AMH captures the difference—underpins the use of AMH as a comparable, cross-species biomarker of reproductive aging, while acknowledging that the mechanistic links between the follicular reserve and adverse pregnancy outcomes remain incompletely defined.

Several limitations should temper the interpretation of these results. Most importantly, the design examined a cohort of old ewes that was homogeneous in both chronological age (six to eight years) and parity, assessed at a single time point, without a younger comparison group and without longitudinal resampling of the same animals over successive years. The study therefore does not trace the trajectory of ovarian aging directly; rather, it demonstrates that inter-individual heterogeneity in the ovarian reserve—the divergence between biological and chronological ovarian age—is already substantial within an aged population and carries measurable reproductive consequences. Confirming the temporal course of ovarian decline will require cross-sectional designs spanning multiple age strata or longitudinal cohorts followed across their reproductive lifespan. The overall sample was also modest (n = 44) and the individual outcome subgroups were small (n = 5–8), which limited statistical power for the within-group correlation analyses and precludes firm mechanistic conclusions from those exploratory tests. Animals originated from a single flock, so the generalizability of the specific associations to other breeds, management systems, and seasons requires confirmation. Ovarian reserve was inferred solely from circulating AMH. No direct structural assessment of the follicle pool, whether by ultrasonographic antral follicle count or by histological quantification, was performed, and this is a substantive limitation rather than a minor omission: the mechanistic argument advanced here, namely that differences in AMH index differences in ovarian reserve which in turn shape reproductive outcome, rests entirely on a single circulating biomarker and was not independently corroborated. A second and related caution concerns what low AMH may be standing proxy for. In aged ewes, a reduced AMH concentration may reflect generally poorer physiological or nutritional condition, or an overall advanced state of reproductive senescence, rather than acting as an independent determinant of fertility. The present design cannot separate these possibilities. Body weight, body condition score and parity did not differ significantly among the outcome groups at enrolment, which argues against gross confounding by condition, but no multivariable model adjusting AMH for these and other covariates was fitted, in part because the size of the individual subgroups (n = 5–8) would not have supported one. Whether AMH carries information about reproductive outcome that is independent of conventional condition and reproductive-history variables therefore remains an open question, and one that a larger cohort analyzed with a multivariable time-to-conception model would be well placed to answer. The absence of breed-standardized, assay-specific reference ranges for ovine AMH also complicates the translation of group differences into actionable thresholds. Finally, the observational design establishes association rather than causation, and the reported relationships between AMH and both conception timing and pregnancy loss will need to be validated prospectively. Because several subgroup analyses were based on small sample sizes (n = 5–8), Spearman correlation coefficients were interpreted primarily as measures of effect size rather than relying solely on statistical significance. Accordingly, the magnitude and direction of the observed associations were considered more informative than the corresponding P values, and these subgroup findings should be regarded as exploratory and hypothesis-generating pending confirmation in larger cohorts.

## Conclusions

In aged multiparous ewes, serum AMH measured at the onset of the breeding season was associated with subsequent conception timing and with pregnancy loss, whereas progesterone and estrogen were not. Because animals were grouped according to outcomes that had already occurred, and because no diagnostic threshold was derived or validated, these findings establish an association rather than a validated predictive test, and they should not yet be used to guide culling decisions in practice. Four specific steps would be required before AMH could be proposed for that purpose. First, prospective validation in larger, multi-breed cohorts recruited across more than one flock and season, with the analysis specified before outcomes are known. Second, formal derivation of a decision threshold with reporting of sensitivity, specificity and the area under the receiver operating characteristic curve, together with confidence intervals, and with a sample size calculated for that purpose rather than for group comparison. Third, incorporation of antral follicle counts, so that the assumed link between circulating AMH and the structural ovarian reserve is measured rather than inferred. Fourth, multivariable modelling, ideally treating the cycle of conception as a time-to-event outcome, to establish whether AMH contributes information beyond body condition, parity and other conventional reproductive variables. Until these steps are taken, the present results are best regarded as a hypothesis-generating basis for such work, and as an incremental contribution to the use of the ewe as a model of ovarian ageing.

## Data Availability

The raw data supporting the conclusions of this article will be made available by the authors, without undue reservation.
